# Coronary Angiography-Derived Index of Microvascular Resistance

**DOI:** 10.3389/fphys.2020.605356

**Published:** 2020-12-16

**Authors:** Hu Ai, Yundi Feng, Yanjun Gong, Bo Zheng, Qinhua Jin, Hui-Ping Zhang, Fucheng Sun, Jianping Li, Yundai Chen, Yunlong Huo, Yong Huo

**Affiliations:** ^1^Department of Cardiology, Beijing Hospital, Beijing, China; ^2^National Center of Gerontology, Beijing, China; ^3^PKU-HKUST Shenzhen-Hong Kong Institution, Shenzhen, China; ^4^Department of Cardiology, Peking University First Hospital, Beijing, China; ^5^Department of Cardiovascular, PLA General Hospital, Beijing, China; ^6^Institute of Mechanobiology & Medical Engineering, School of Life Sciences & Biotechnology, Shanghai Jiao Tong University, Shanghai, China

**Keywords:** index of microcirculatory resistance, instantaneous wave–free ratio (IFR), fractional flow reserve (FFR), computational fluid dynamics (CFD), hemodynamics

## Abstract

A coronary angiography-derived index of microvascular resistance (caIMR) is proposed for physiological assessment of microvasular diseases in coronary circulation. The aim of the study is to assess diagnostic performance of caIMR, using wire-derived index of microvascular resistance (IMR) as the reference standard. IMR was demonstrated in 56 patients (57 vessels) with stable/unstable angina pectoris and no obstructive coronary arteries in three centers using the Certus pressure wire. Based on the aortic pressure wave and coronary angiograms from two projections, the caIMR was computed and assessed in blinded fashion against the IMR at an independent core laboratory. Diagnostic accuracy, sensitivity, specificity, positive predictive value and negative predictive value of the caIMR with a cutoff value of 25 were 84.2% (95% CI: 72.1% to 92.5%), 86.1% (95% CI: 70.5% to 95.3%), 81.0% (95% CI: 58.1% to 94.6%), 88.6% (95% CI: 76.1% to 95.0%), and 77.3% (95% CI: 59.5% to 88.7%) against the IMR with a cutoff value of 25. The receiver-operating curve had area under the curve of 0.919 and the correlation coefficient equaled to 0.746 between caIMR and wire-derived IMR. Hence, caIMR could eliminate the need of a pressure wire, reduce technical error, and potentially increase adoption of physiological assessment of microvascular diseases in patients with ischemic heart disease.

## Introduction

Microvascular diseases have recently shown an increase in importance for the diagnosis and management of patients with chronic coronary syndrome (Kaski et al., 2018; Ford et al., 2020a; Knuuti et al., 2020; Kunadian et al., 2020). The pathological mechanisms for coronary microvascular diseases are heterogeneous (Huo and Kassab, 2009; Huo et al., 2009; Lanza, 2019). Microvascular angina (MVA) features patients with normal coronary arteries and evidence of myocardial ischemia owing to coronary microvascular dysfunctions (Cannon and Epstein, 1988; Kunadian et al., 2020). This type of patients constitute >20% patients in the cardiac catheterization laboratory (Ford et al., 2018, 2020b). Although multiple non-invasive methods, such as PET and cardiac MRI, were suggested for assessment of the MVA (Kunadian et al., 2020), invasive methods are considered the gold standard. Fearon and his colleagues proposed an index of microcirculatory resistance (IMR) to quantify microcirculatory dysfunctions in patients with ischemic heart disease (IHD) (Fearon et al., 2003, 2004; Aarnoudse et al., 2004), the measurements of which by a thermodilution wire in the maximal hyperemia were assumed to be remarkably reproducible as compared with other hemodynamic parameters (e.g., coronary flow reserve-CFR, hyperemic myocardial resistance-HMR, and hyperemic stenosis resistance-HSR) (Fearon et al., 2004; Pagonas et al., 2014).

Physiological parameters, fractional flow reserve (FFR) (Pijls et al., 2007, 2010; Tonino et al., 2009; van Nunen et al., 2015) and adenosine-free instantaneous wave–free ratio (iFR) (Davies et al., 2017; Gotberg et al., 2017), have been strongly suggested to guide the decision-making revascularization for epicardial stenoses that are not a sole cause of IHD (Lee et al., 2015). Lee et al. (2015) suggested a combination of IMR and FFR to show the relative contribution of macro- and microvascular diseases in patients with IHD. In recent years, coronary angiography-derived FFR without using invasive pressure-wire measurement and hyperemic stimulus has shown high diagnostic accuracy by using wire-derived FFR as the reference standard (Xu et al., 2017; Fearon et al., 2019; Li et al., 2020). Here, we propose a novel coronary angiography-derived index of microvascular resistance (caIMR) (see the definition in the Appendix A). There is, however, lack of clinical validation for the caIMR.

The objective of the study is to evaluate diagnostic performance of the caIMR using wire-derived IMR as the reference standard. This study retrospectively analyzed patients with stable/unstable angina pectoris and no obstructive coronary arteries in three hospitals at Beijing, China. The computed caIMR was compared with the measured IMR in these patients. The significance and implications of the study were discussed relevant to adenosine-free indexes of coronary physiology.

## Materials and Methods

### Theory

**FIGURE 1:**
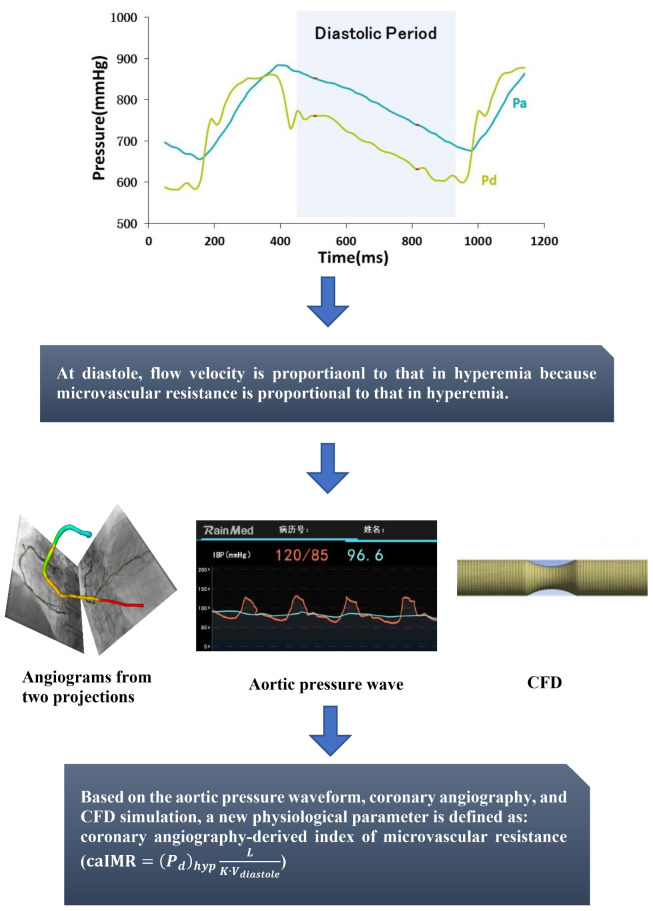
Schematic representative performance of caIMR.

Based on the aortic pressure wave and coronary angiograms from two projections, a novel physiological parameter, caIMR (unit: mmHg⋅s/mm), is proposed as follows:

(1) $$\text{caIMR}={\left(P_{d}\right)}_{hyp}\frac{L}{K\cdot V_{diastole}}$$

where *L* is a constant (non-dimentional) that mimics the length from the inlet to the distal position (*L=75*, mimicking 75 mm downstream from the inlet of coronary arterial tree), (*P*_*d*_)*hyp* is the mean pressure (unit: mmHg) at the distal position at the maximal hyperemia, *V*_*diastole*_ is the mean flow velocity (unit: mm/s) at the distal position at diastole, and *K* is a constant (*K* = 2.1) obtained from a previous literature (Johnson et al., 2013) and *V*_*hyp*_ = *K*⋅*V*_*diastole*_ refers to the mean flow velocity (unit: mm/s) at the distal position at the maximal hyperemia, as shown in Figure 1. Here, caIMR characterizes the microvascular resistance in unit volume of myocardium distal to the *L* position. The detailed theoretical derivation is described in the Appendix A.

### Clinical Study

The retrospective trial was demonstrated to assess diagnostic performance (e.g., feasibility, accuracy and safety) of the caIMR in the FlashAngio system (including the FlashAngio console, FlashAngio software and FlashPressure pressure transducer; Rainmed Ltd., Suzhou, China). The retrospective clinical trial was approved by the Institutional Review Boards (IRB) in Beijing Hospital, PLA General Hospital and Peking University First Hospital at Beijing, China, which conforms the declaration of Helsinki and Good Clinical Practice Guidelines of the China Food and Drug Administration. Written consent was waived owing to the minimal patient risk in accordance with the relevant guidelines and regulations of the IRB from the three centers.

### Participants

Patients (aged at least 18 years) with stable or unstable angina pectoris and no obstructive coronary arteries by angiographic visual estimation (i.e., area stenoses <50% from the observation of the interventional cardiologist) were enrolled in Beijing Hospital, PLA General Hospital and Peking University First Hospital at Beijing, China. Participants were excluded if they had suffered myocardial infarction within previous 7 days; had left ventricular ejection fraction ≤ 50%; estimated glomerular filtration rate (eGFR) < 45 ml/min (or 1.73 m^2^); had known severe coagulopathy or bleeding disorders; were allergic to iodine contrast agents, adenosine or ATP. Angiographic exclusion criteria included poor contrast opacification, severe vascular overlap or distortion of the interrogated vessel, or when poor angiographic image quality precluded contour detection required by the FLASH software.

### Procedures

Coronary angiography from multiple views, at the operators’ discretion, was recorded at 15 or 30 frames per second. For computation of the caIMR, contrast was injected with standard manual force to opacify the coronary arterial tree. At least two angiographic projections avoiding vessel overlap, separated by ≥ 30^*o*^, without table movement, were required to compute the caIMR (Li et al., 2020). The measured aortic pressure wave during the wire-derived IMR measurement and Digital Imaging and Communications in Medicine (DICOM) angiography images were input to the FlashAngio console. A three-dimensional (3D) mesh reconstruction of coronary arteries was generated along the vessel path from the inlet to the most distal position. Mean aortic pressure (MAP) were computed by averaging the pressure waves in three cardiac cycles, based on which the maximal hyperemic MAP, (*P*_*a*_)*hyp*, is determined using the mathematical formula in the Appendix of a previous study (Li et al., 2020).

The diastolic flow velocity (*V*_*diastole*_) was determined automatically by the FlashAngio software, similar to a previous study (Gong et al., 2020). Briefly, based on the movement of the tip of the guiding catheter (direct connecting to the coronary arterial tree) in angiograms, we can determine systolic and diastolic periods, where the shorter time interval refers to the systolic period and the longer time interval represents the diastolic period as the tip of guiding catheter moves in or out. We compute the diastolic flow velocity by the Thrombolysis in Myocardial Infarction (TIMI) Frame Count Method (Gibson et al., 1996; Dodge et al., 1998), i.e., diastolic flow velocity = (contrast passing length)/(diastolic time interval), where contrast passing length is the distance that contrast moves in 3D reconstructed coronary arteries during the period of diastole. The maximal hyperemic flow velocity, *V*_*hyp*_, is assumed equal to 2.1×*V*_*diastole*_ (Johnson et al., 2013).

We have developed a specially designed CFD model to carry out the steady-state laminar flow simulation across the stenotic blood vessel in 10–30 s (Li et al., 2020), which is described in Appendix B. The CFD method with the inlet velocity of *V*_*hyp*_ was used to solve Navier-Stokes and continuity equations in the FlashAngio software and compute the pressure drop [(Δ*P*)*hyp*] along meshed coronary arteries from the inlet to the distal position (*L* = 75*mm* downstream from the inlet of coronary arterial tree) and (*P*_*d*_)*hyp* = (*P*_*a*_)*hyp*−(Δ*P*)*hyp*. The caIMR was computed in equation [1] by some researchers in an independent core lab blinded to the wire-derived IMR measurement.

### Wire-derived IMR Measurement

A Certus pressure wire (St. Jude Medical, St. Paul, MI, United States) was inserted to the distal position (i.e., the same position in the FlashAngio software) by interventional cardiologists. Intracoronary nitrate (100 μg) was administered before physiological measurements. Hyperemic blood flow was induced by IV administration of adenosine-5′-triphosphate (ATP) at ≥140 μg/kg/min. To derive mean transit time (T_*mn*_), thermodilution curves were obtained by at least 3 injections of 3–4 ml of 4^*o*^ C saline during sustained hyperemia. Performance of the wire-derived IMR measurement was according to the standard procedures suggested by the RadiAnalyzer Xpress instrument (St. Jude Medical, St. Paul, MI, United States). Moreover, examination of pressure drift was carried out through a pull-out of the pressure wire to the guiding catheter tip, where the ratio of (wire-derived mean pressure)/MAP should be between 0.97 and 1.03.

### Statistical Analysis

Baseline demographics of all patients were recorded as mean ± standard deviation (SD) or percentage with counts. Diagnostic accuracy, sensitivity, specificity, positive predictive value (PPV), and negative predictive value (NPV) (Trevethan, 2017) of the caIMR were calculated with wire-derived IMR as the reference standard. Two-sided 95% confidence intervals (CIs) were added to these parameters using the Clopper-Pearson exact method. Receiver-operating curves of the caIMR, with the IMR as the gold standard, were estimated by using the logistic regression model. The cutoff value of 25 was applied to the IMR (Ford et al., 2020a; Kunadian et al., 2020) and receiver-operating curves were used to find a good correlation between caIMR and IMR. All statistical analyses were performed with a test significance level of 0.05.

## Results

Wire-derived IMR and caIMR were successfully demonstrated in 56 patients (57 vessels) with no obstructive coronary arteries at the age of 61.9 ± 9.2 years (53.6% male). Baseline patient characteristics are presented in Table 1. The predominant patient presentation is stable angina pectoris (62.5%) and unstable angina pectoris (32.1%). There are no prior myocardial infarctions in all patients. Offline caIMR computations are carried out in these patients at an independent laboratory in blinded fashion. The mean values of IMR and caIMR equal to 37.1 ± 22.1 and 35.5 ± 17.4, respectively, showing no statistical difference.

**TABLE 1:** Baseline characteristics of the study population.

Baseline Characteristics	*n* = 56
Age (year)	61.9 ± 9.2
Male	30 (53.6%)
BMI	27.1 ± 4.0
LV ejection fraction (%)	65.9 ± 3.2
Systolic blood pressure (mmHg)	132 ± 13
Diastolic blood pressure (mmHg)	78 ± 12
Hypertension	30 (53.6%)
Hyperlipidemia	37 (66.1%)
Diabetes mellitus	28 (50.0%)
Current smoking	16 (28.6%)
Prior PCI	3 (5.4%)
Prior CABG	None
Prior myocardial infarction	None
Silent ischemia	3 (5.4%)
Stable angina pectoris	35 (62.5%)
Unstable angina pectoris	18 (32.1%)
Acute myocardial infarction within 1 months	None

*Values are in n (%) or mean ± standard deviation.*

**FIGURE 2:**
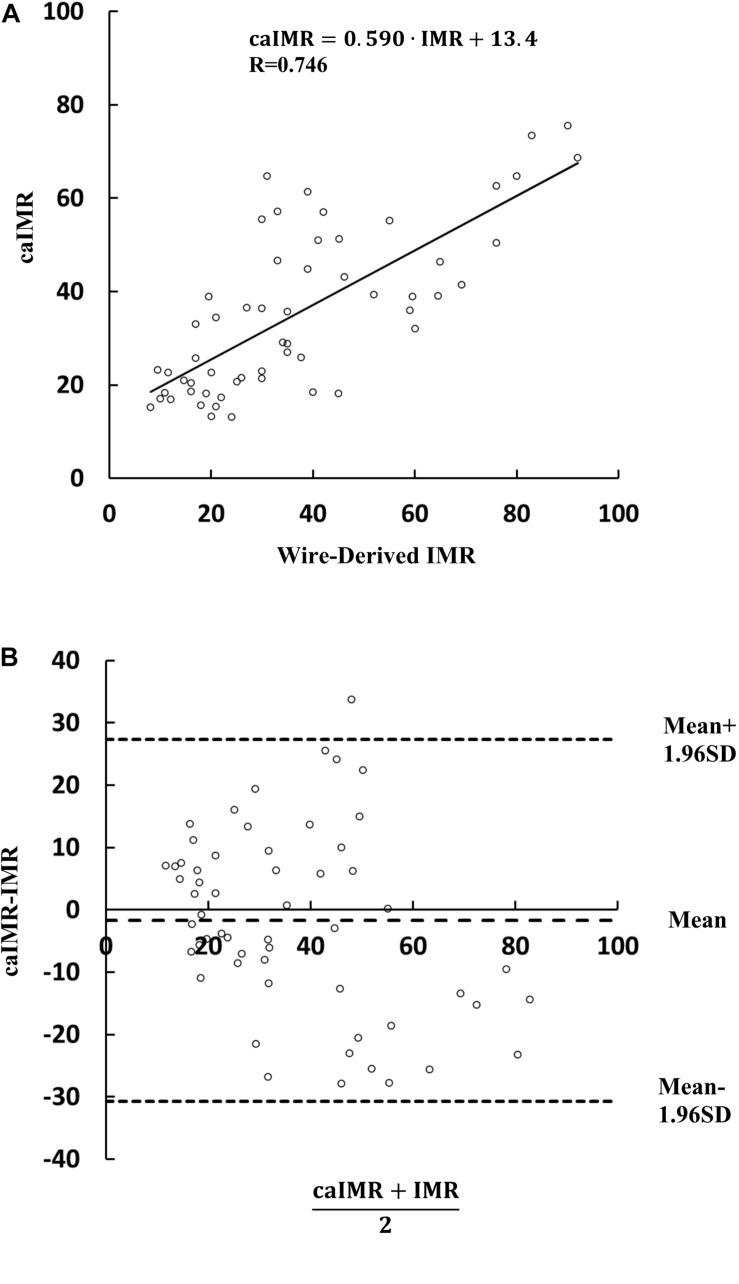
Correlation and agreement between wire-derived IMR and caIMR. **A** A least-squares fit shows a relationship: caIMR = 0.590⋅IMR + 13.4 (R = 0.746) (Vessel Number: *n* = 57) and **(B)** Bland-Altman plots for pairwise comparisons (mean difference: −1.68; SD: 14.8; 95% limits of agreement −30.7 to 27.4) (Vessel Number: *n* = 57). The student’s *t*-test shows *p* value of 0.654 (Vessel Number: *n* = 57).

**FIGURE 3:**
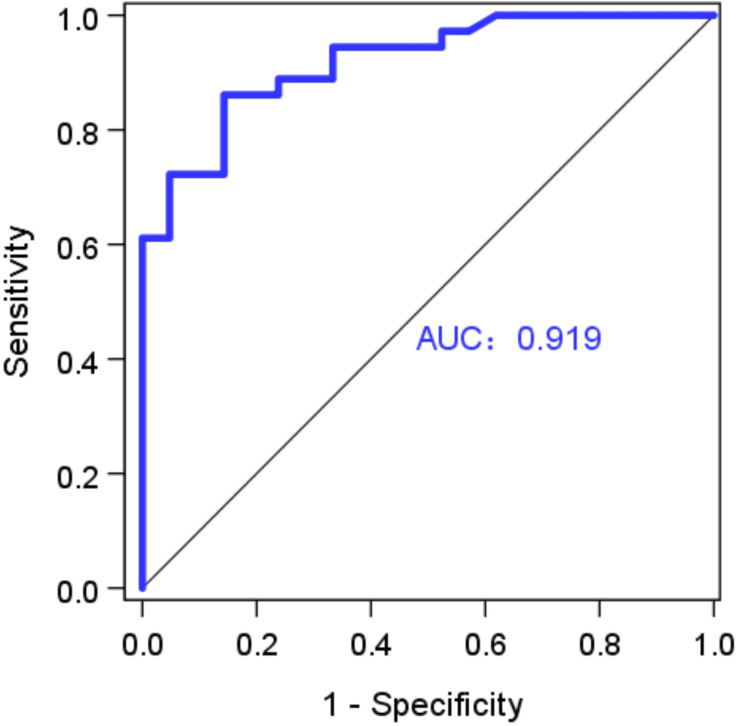
Receiver-operating curve for wire-derived IMR (a cutoff of 25) and caIMR (a cutoff of 25), where AUC (area under the curve) is 0.919 (Vessel Number: *n* = 57).

Figure 2A shows the linear relationship between caIMR and wire-derived IMR (caIMR = 0.590⋅IMR + 13.4, R = 0.746). Bland-Altman analysis did not identify systematic differences between caIMR and IMR, with a mean difference of −1.68 ± 14.8 (95% limits of agreement −30.7 to 27.4, Figure 2B). Table 2 lists diagnostic performance of the caIMR for 57 vessels by using wire-derived IMR as the standard reference with a cutoff value of 25. The caIMR with a cutoff value of 25 has the highest receiver-operating characteristic AUC. The caIMR has diagnostic accuracy, sensitivity, specificity, positive predictive value (PPV), and negative predictive value (NPV) of 84.2% (95% CI: 72.1% to 92.5%), 86.1% (95% CI: 70.5% to 95.3%), 81.0% (95% CI: 58.1% to 94.6%), 88.6% (95% CI: 76.1% to 95.0%), and 77.3% (95% CI: 59.5% to 88.7%) against the wire-derived IMR. Accordingly, Figure 3 shows the receiver-operating characteristic AUC of 0.919.

**TABLE 2:** Diagnostic characteristics of caIMR using the wire-derived IMR as standard reference in 57 vessels.

caIMR cutoff = 25; IMR cutoff = 25	AUC = 0.919 [0.851; 0.987]
Diagnostic accuracy	84.2% [72.1%; 92.5%]
Sensitivity	86.1% [70.5%; 95.3%]
Specificity	81.0% [58.1%; 94.6%]
Positive Predictive Value	88.6% [76.1%; 95.0%]
Negative Predictive Value	77.3% [59.5%; 88.7%]
Disease prevalence	63.2% [49.3%; 77.6%]

## Discussion

This retrospective study assessed the caIMR against the wire-derived IMR in 56 patients (57 vessels). We reported that caIMR (a cutoff value of 25) showed the correlation coefficient of R = 0.746 (receiver-operating characteristic AUC of 0.919) with wire-derived IMR (a cutoff value of 25) in patients with stable/unstable angina pectoris and no obstructive coronary arteries.

Substantial clinical trials have shown that FFR improved patient outcomes and led to significant resource savings in patients with stenoses (Fearon et al., 2010). Since intracoronary pressure and flow decline in a linear fashion at the wave-free period (WFP) of the diastole (Hoffman and Spaan, 1990), microvascular resistance is stable and lower than that over the rest of a cardiac cycle, but higher than that at the maximal hyperemia (Sen et al., 2012). Hence, adenosine-free iFR was defined in the WFP of the diastole to feature physiological impact of coronary stenoses on the distal coronary bed without the confounding influences of myocardial contraction on coronary circulation (Sen et al., 2012), showing a non-inferior revascularization strategy to a FFR-guided strategy with respect to the rate of major adverse cardiac events at 1 year (Davies et al., 2017; Gotberg et al., 2017). Moreover, diastolic pressure ratio (dPR) offered numerical equivalency to iFR (Johnson et al., 2019) such that the entire period of diastole had the same physiological characteristics in the distal coronary bed as the WFP of the diastole. Here, we assumed that the mean flow velocity in the entire period of diastole is approximately proportional to that in hyperemia, i.e., *V*_*hyp*_ = *K*⋅*V*_*diastole*_ and *K*≈ 2.1 obtained from Table T10 in a previous study (Johnson et al., 2013). This model was applied to computation of caIMR albeit significant improvement are still required in future studies.

In comparison with FFR-, iFR- and dPR-guided revascularization for epicardial stenoses, the thermodilution-derived IMR was applied to the diagnosis of microcirculatory diseases in patients with different manifestations, e.g., patient has abnormal stress study and angina but has no significant epicardial coronary lesions; patient has undergone successful PCI but continues to have angina; patient presenting with acute coronary syndrome; and patient after heart transplantation (Lee et al., 2015; Ahn et al., 2016; Kobayashi et al., 2017). Although HMR and HSR were proposed for microcirculation assessment, there was still lack of data about their clinical values in guiding revascularization given less used doppler evaluation in a combo-pressure-velocity-wire. On the other hand, Fearon and his colleagues have confirmed the IMR as a quantitative assessment of the hyperemic microvascular resistance independent of the FFR and supported IMR-guided interventions of microcirculation by using a pressure–temperature sensor guidewire (Martinez et al., 2015). The wire-free and CFD-derived caIMR was first defined in Eq. [1] of the present study for physiological assessment of microcirculatory dysfunction, based on the measured aortic pressure wave and TIMI-frame count-based flow velocity during the entire diastolic period. The caIMR could be associated with the IMR (Johnson et al., 2016).

The wire-derived IMR was found to be < 25 in a control group without evidence of atherosclerosis (Melikian et al., 2010). The high IMR was defined as ≥ 25, i.e., ≥ 75^*th*^ percentile in 1096 patients with major coronary arteries from 8 centers in 5 countries (Lee et al., 2015). Hence, the cutoff value of IMR was assumed equal to 25 in patients of ischemia with non-obstructive coronary arteries (INOCA) according to the EAPCI Expert Consensus Document in Collaboration with European Society of Cardiology Working Group published in 2020 (Kunadian et al., 2020). In the retrospective study, the caIMR with a cutoff value of 25 has shown diagnostic accuracy, sensitivity and specificity of 84.2%, 86.1%, 81.0%, respectively, by using wire-derived IMR as the standard reference with a cutoff value of 25 (AUC of 0.919). This shows reasonable agreement between caIMR and wire-derived IMR.

On the other hand, Fearon et al. have measured IMR immediately after PCI in 253 patients with acute coronary syndrome from 3 centers. A cutoff of 40 measured immediately after PCI was suggested given higher 1-year rates of death or hospitalization (Fearon et al., 2013). Hence, patients with INOCA have different cutoff value of IMR from patients with acute coronary syndrome (25 vs. 40), the application to whom requires more validations.

## Limitations

This study only compared caIMR with the measured IMR in patients with INOCA by angiographic visual estimation (area stenoses <50%). Patients have undergone successful PCI but continue to have angina, patients with acute coronary syndrome, and patients after heart transplantation should be considered in the following studies. On the other hand, patients with vascular reactivity related to abnormalities in endothelial function (vasospastic angina) were recommended to take the intracoronary acetylcholine test (Kunadian et al., 2020).

The retrospective study only included 56 patients (57 vessels) to test the proposed caIMR. The sample size was relatively small. We also used the measured aortic pressure wave during the wire-derived IMR measurement. There were some discordances between caIMR and IMR. IMR was measured in the maximal hyperemia while caIMR was computed based on the angiography-derived diastolic flow velocity in the contrast-induced sub-hyperemia, which is the main reason for the discordances between caIMR and IMR. A comparison of FFR (in the maximal hyperemia) and iFR (WTP period of the diastole at the baseline) has shown some discordances, but iFR showed a non-inferior revascularization strategy to the FFR with respect to the rate of major adverse cardiac events at ≥12 months. This study is still at an early stage of development in angiography-derived microvascular diagnosis and no outcome studies have been performed. The following prospective study in multiple centers should be demonstrated to compare the rates of major adverse cardiac events at ≥12 months between caIMR and IMR in a large cohort of patients when the aortic pressure is measured by using a specialized pressure transducer (FlashPressure, Rainmed Ltd., Suzhou, China) connected to the guiding catheter to record the aortic pressure wave during the entire procedure. A combination of caFFR (Li et al., 2020) and caIMR should also be performed for physiological assessment of patient macro- and microvascular diseases.

## Conclusion

A novel parameter, caIMR, was proposed to characterize physiological impact of the microcirculatory dysfunction. There is correlation coefficient of R = 0.746 between caIMR and wire-derived IMR in patients with INOCA. The caIMR for physiological assessment of the microvascular diseases could provide important insights to patients with IHDs, which requires further investigations.

## Data Availability Statement

The raw data supporting the conclusions of this article will be made available by the authors, without undue reservation.

## Ethics Statement

The studies involving human participants were reviewed and approved by the retrospective clinical trial was approved by the Institutional Review Board (IRB) in Beijing Hospital, PLA General Hospital and Peking University First Hospital, which conforms the declaration of Helsinki and Good Clinical Practice Guidelines of the China Food and Drug Administration. The patients/participants provided their written informed consent to participate in this study.

## Author Contributions

HA, H-PZ, BZ, QJ, FS, and YC performed the experimental measurement. YF and YG performed the theoretical analysis. YLH drafted the manuscript. YLH, JL, and YH reviewed the manuscript. All authors contributed to the article and approved the submitted version.

## Conflict of Interest

YH holds stocks of Rainmed Ltd., Suzhou, China. HA is supported by the Beijing Hospital Clinical Research 121 Project (BJ-2019-193); and there is nothing to disclose for others.

## Appendix A

Similar to the wave-free period (WFP) of the diastole (Sen et al., 2012; Nijjer et al., 2016), the entire diastolic period provides higher flow velocity and results in lower microvascular resistance than the whole cardiac cycle at rest (Hoffman and Spaan, 1990). Moreover, an IC injection of contrast medium can reasonably induce some degree of hyperemia (Johnson et al., 2016) albeit an IV injection of adenosine leads to the maximal hyperemia. We assume that the flow velocity at diastole (*V*_*diastole*_, unit: mm/s) is proportional to that in hyperemia (*V*_*hyp*_, unit: mm/s), i.e., *V*_*hyp*_ = *K*⋅*V*_*diastole*_ and *K* is a constant [*K*≈ 2.1 obtained from Table T10 in a previous study (Johnson et al., 2013)]. Hence, we propose a novel physiological parameter, caIMR (unit: mmHg⋅s/mm), as:

(A1) $$\text{caIMR}={\left(P_{d}\right)}_{hyp}\frac{L}{V_{hyp}}={\left(P_{d}\right)}_{hyp}\frac{L}{K\cdot V_{diastole}}$$

where *L* is a constant that mimics the length from the inlet to the distal position (*L=75*, mimicking 75 mm downstream from the inlet of coronary arterial tree) and (*P*_*d*_)*hyp* is the mean pressure (unit: mmHg) at the distal position at the maximal hyperemia, which is computed by the FlashAngio software.

Fearon et al. proposed the IMR to assess the microcirculation using the thermodilution method measured by a pressure–temperature sensor guidewire (Fearon et al., 2003) as:

(A2) $$\text{IMR}={\left(P_{d}\right)}_{hyp}\cdot T_{mn}\;\mathrm{at}\mathrm{the}\mathrm{maximal}\mathrm{hyperemia}$$

where (*P*_*d*_)*hyp* is the mean pressure (unit: mmHg) at the distal position (∼75 mm downstream from the inlet of coronary arterial tree) and *T*_*mn*_ is the mean transit time (unit: s) at the maximal hyperemia, which are measured by the pressure–temperature sensor guidewire. On the other hand, Meuwissen et al. (2001) defined the hyperemic myocardial resistance-velocity based index (HMR, unit: mmHg⋅s/cm given that the unit of *V*_*hyp*_ in HMR is cm/s) to evaluate microvascular dysfuctions as:

(A3) $$\text{HMR}=\frac{{\left(P_{d}\right)}_{hyp}}{V_{hyp}}\;\mathrm{at}\mathrm{the}\mathrm{maximal}\mathrm{hyperemia}$$

To assess the microcirculation, we propose caIMR (= HMR×*L*). Since $T_{mn}\propto \frac{1}{V_{hyp}}$, there should have a proportional relationship between caIMR and IMR and HMR.

## Appendix B

A CFD method was applied to solving the equations of continuity and Navier-Stokes as:

(B1) $$\nabla \cdot \hat{V}=0$$

(B2) $$\rho \frac{\partial \hat{V}}{\partial t}+\rho \hat{V}\cdot \nabla \hat{V}=-\nabla P+\nabla \cdot \mu \left(\nabla \hat{V}+{\left(\nabla \cdot \hat{V}\right)}^{T}\right)$$

where $\hat{V}$, *P*, ρ, and μ represent the velocity, pressure, blood mass density, and viscosity, respectively. The inlet boundary condition is the hyperemic flow velocity, *V*_*hyp*_ = *K*⋅*V*_*diastole*_. The pressure drop, (Δ*P*_*s*_)*hyp*, across a stenosis is computed from the CFD simulation. If the interval between the centers of two serial stenoses <3 cm, the pressure drops across them are computed together. We generated a database including thousands of various pipe flows by using the previously validated finite element model (Huo and Li, 2004). The database took account of the changes of the inlet flow velocity, stenotic diameter and length, inlet and outlet of a stenosis, and curvature of a stenosis. Based on the database, we optimized the initial condition for various cases to significantly reduce the iterations of the convective term in the steady-state laminar flow simulation. Moreover, we significantly reduced the meshes of the control volume to ensure the relative error < 2% between dense and sparse meshes. The specifically designed CFD solver can fast and accurately compute the pressure drop in the steady-state laminar flow simulation. The pressure drop, (Δ*P*)*hyp*, along the meshed coronary arteries in the vessel path from the inlet to the most distal position was:

(B3) $${\left(\Delta P\right)}_{hyp}=\sum {\left(\Delta P_{s}\right)}_{hyp}$$

The computational time of CFD simulation is 10–30 s.
